# Phospholipid composition and kinetics in different endobronchial fractions from healthy volunteers

**DOI:** 10.1186/1471-2466-14-10

**Published:** 2014-02-01

**Authors:** Ahilanandan Dushianthan, Victoria Goss, Rebecca Cusack, Michael PW Grocott, Anthony D Postle

**Affiliations:** 1 NIHR Respiratory Biomedical Research Unit, University Hospital Southampton NHS Foundation Trust, Southampton SO16 6YD, UK; 2 Integrative Physiology and Critical Illness Group, Clinical and Experimental Sciences, Sir Henry Wellcome Laboratories, Faculty of Medicine, University of Southampton, Southampton SO16 6YD, UK; 3 Anaesthesia and Critical Care Research Unit, CE 93, MP24, E-Level, Centre Block, University Hospital Southampton NHS Foundation Trust, Southampton SO16 6YD, UK

**Keywords:** Surfactant, Phosphatidylcholine, Deuteriated choline, Stable isotopes, Isotope labelling, Mass spectrometry

## Abstract

**Background:**

Alterations in surfactant phospholipid compositions are a recognized feature of many acute and chronic lung diseases. Investigation of underlying mechanisms requires assessment of surfactant phospholipid molecular composition and kinetics of synthesis and turnover. Such studies have recently become possible in humans due to the development of stable isotope labelling combined with advances in analytical methods in lipidomics. The objectives of this study are to compare phospholipid molecular species composition and phosphatidylcholine synthesis and turnover in surfactant isolated from various endobronchial compartments in healthy adults.

**Methods:**

Healthy adults (N = 10) were infused with *methyl*-D_9_-choline chloride and samples of induced sputum, tracheal wash and small volume bronchoalveolar lavage fluid were obtained subsequently at intervals up to 96 hours. Surfactant phospholipid composition and incorporation of stable isotope into surfactant phosphatidylcholine were determined by electrospray ionisation mass spectrometry.

**Results:**

While molecular species compositions of phospholipids were similar for all three sample types, dipalmitoylphosphatidylcholine content was highest in lavage, intermediate in tracheal wash and lowest in sputum. *Methyl*-D_9_-choline incorporation into surfactant phosphatidylcholine was lower for sputum at 24 hours but reached equilibrium with other sample types by 48 hours. Fractional *methyl*-D_9_-dipalmitoylphosphatidylcholine incorporation for all sample types was about 0.5% of the endogenous composition. Lysophosphatidylcholine enrichment was twice than that of phosphatidylcholine.

**Conclusions:**

Tracheal secretions may be of value as a surrogate to assess bronchoalveolar lavage fluid surfactant molecular composition and metabolism in healthy people. Despite minor differences, the phospholipid molecular composition of induced sputum also showed similarities to that of bronchoalveolar lavage fluid. Detailed analysis of newly synthesized individual phosphatidylcholine species provided novel insights into mechanisms of surfactant synthesis and acyl remodelling. Lysophosphatidylcholine *methyl*-D_9_ incorporation patterns suggest that these species are secreted together with other surfactant phospholipids and are not generated in the air spaces by hydrolysis of secreted surfactant phosphatidylcholine. Application into patient populations may elucidate potential underlying pathophysiological mechanisms that lead to surfactant alterations in disease states.

## Background

Continued synthesis and secretion of pulmonary surfactant is critically important for maintenance of optimal lung function throughout life. While primary surfactant deficiency in the lungs of preterm infants is widely acknowledged as a major cause of neonatal respiratory distress syndrome, secondary surfactant deficiency contributes to the pathology of many respiratory disorders of the mature lung including acute lung injury (ALI)/ acute respiratory distress syndrome (ARDS), asthma and cystic fibrosis [1]. Described mechanisms for surfactant dysfunction in respiratory diseases include inhibition by plasma proteins such as fibrinogen in oedema fluid, impaired synthesis and secretion by type II alveolar epithelial (ATII) cells and hydrolysis or oxidation of secreted surfactant [2]. Differentiating between these potential mechanisms is problematical in the clinical setting. Acquiring samples of alveolar and/or bronchial secretions for analysis of surfactant function or composition typically involves invasive bronchoscopic and bronchoalveolar lavage (BAL) procedures [3]. Additionally, analysis of concentrations and compositions of surfactant components such as surfactant proteins or phospholipids provides no information about processes of surfactant synthesis, secretion or turnover [4,5]. Availability of such information has considerable potential clinical implications, both for better understanding mechanisms of lung disease and for optimizing treatments for individual patients. Consequently, in this study we have assessed the molecular specificity of surfactant phospholipids extracted from small volume bronchoalveolar lavage fluid (BALF), tracheal wash (TW) and induced sputum (IS), representing secretions from various endobronchial compartments. In addition, we employed an *in vivo* stable isotope labelling strategy to monitor the incorporation of *methyl*-D_9_-choline chloride into the phosphatidylcholine (PC) and lysophosphatidylcholine (LPC) fractions of the three different sample types. PC, especially the disaturated dipalmitoyl species (PC16:0/16:0), is the major surface active component of surfactant phospholipid. This stable isotope methodology enables the assessment of both the rate of synthesis and secretion of individual molecular species of surfactant PC and the specificity of fatty acyl remodelling mechanisms involved in their synthesis. This detailed analysis not only provides important information about mechanisms of surfactant PC synthesis and secretion, but comparison of fractional incorporation rates between sample types can also demonstrate the time required for newly secreted alveolar surfactant to transit to the upper airways.

## Methods

### Materials

*Methyl*-D_9_-choline chloride was from Cambridge Isotopes (CK Gases, Ibstock, UK); dimyristoylphosphatidylcholine (PC14:0/14:0), heptadecyllysophosphatidylcholine (LPC17:0) and dimyristoylphosphatidylglycerol (PG14:0/14:0) were from Avanti Polar Lipids (Alabaster, USA). Solvents of HPLC quality from Fisher Scientific, UK.

### Study population

Ten healthy volunteers without pre-existing lung diseases were recruited. All were non-smokers and had a normal medical examination including spirometry. Subjects with recent (within and up to 4 weeks) history of upper or lower respiratory tract infections were excluded. The age range was 18–36 with a median age of 26. The study protocol was approved by national ethics committee (South Central, Berkshire 11/SC/0185) and the University Hospital Southampton Research and Development Department. Informed consent was obtained from all healthy volunteers prior to the enrolment.

### Methyl- D_9_ choline chloride

Choline is an essential nutrient and a major constituent of the phospholipid fraction of pulmonary surfactant. Deuteriated choline (*methyl*-D_9_ choline chloride) is a stable isotope of choline, which can be used to trace phospholipid synthetic pathways. Recruited volunteers were intravenously infused with *methyl*-D_9_ choline chloride (3.6 mg/kg body weight) for a period of 3 hours in accordance with a previous protocol [6].

### Induced sputum, tracheal wash and small volume BALF

Sputum was induced by nebulised 4.5% hypertonic saline [7]. The induction was performed up to 20 minutes and stopped after sufficient material was obtained (~2 mls). There were no events of bronchospasm or significant drop (>15%) in peak flow during or after the process. Induced sputum was immediately mixed with 5 mls of phosphate buffered saline (PBS) and transferred to ice. Tracheal wash and small volume broncholaveolar lavage samples were obtained by a fibre-optic bronchoscope performed under local anaesthesia without pre-medication. The bronchoscope was passed through mouth with the application of topical lidocaine (maximum of 4 mls 2% w/v above vocal cords and 8 mls 1% w/v below vocal cords. Ten mls of warmed saline (37°C) was applied at the distal end of a right lower lobe distal segmental bronchus and was suctioned. A greater than 50% recovery was deemed to be adequate and if there was < 50% recovery, then a further 10 mls of warmed saline was applied. TW samples were obtained by flushing the trachea with 10mls warmed saline with subsequent suction. The subsequent BALF sampling was performed from a left lower lobe distal segmental bronchus next day. There was no significant decline in the FEV_1_ noted after the bronchoscopy. IS, BALF and TW samples were filtered through a 100 μm mesh cell strainer (BD Falcon) and centrifuged at 400 × g ×10 minutes at 4°C to remove cells and debris. The supernatant was aspirated and stored at -80°C.

### Phospholipid extraction

Internal standards of 1 nmol dimyristoyl-PC (PC14:0/14:0), 0.1 nmol heptadecyl_LPC (LPC17:0), 0.2 nmol of dimyristoyl-PG (PG14:0/14:0) were added to all samples. Phospholipid fraction was extracted by modified Bligh and Dyer method [8]. Briefly, 800 μl of sample and the addition of chloroform: methanol: water (v/v 2:2:1) resulted in biphasic layer. The lower phospholipid rich layer was aspirated carefully and dried under nitrogen gas at 37°C.

### Mass spectrometry analysis

Phospholipids were analyzed using a Xevo triple quadrupole mass spectrometer with electrospray ionisation interface (Walters, UK). Dried lipid extracts were dissolved in methanol:butanol:water:25%NH_4_ (6:2:1.6:0.4 v/v) and delivered by direct infusion at 8 μl/min. PC, LPC and sphingomyelin (SPH) species were identified by precursor scans of the phosphocholine head group fragment, quantifying endogenous PC and LPC species from the fragment of mass to charge ratio (m/z) +184 and deuteriated PC and LPC species from the m/z +193 fragment. Phosphatidylglycerol (PG) and phosphatidylinositol (PI) species were quantified from the negative ionisation spectrum. The ion peaks were quantified using MassLynx software with an in-house Excel macro programmed in Visual Basic.

### Determination of SP-D

The SP-D content was determined by enzyme- linked immunosorbent assay (ELISA). The plates (96-well Nunc MaxiSorp, Fisher Scientific) were coated with capture antibody rfhSP-D 1ug/100ul per well in carbonate binding buffer (Sigma-Aldrich) and incubated at 4° overnight. Plates were washed three times (PBS/T 0.05% (v/v) Tween 20) and blocked for one hour (PBS/T with 2% BSA) at room temperature. After further wash, samples and standards were incubated for one hour at room temperature. After washing again the plates were incubated with streptavidin horseradish peroxidise 1:10,000 (Sigma-Aldrich) for one hour at room temperature. The plates were developed with TMB (Sigma-Aldrich), reaction was stopped with 0.5 M H_2_S0_4_ and plates were read at 450 nm.

### Statistics

The data are expressed as mean ± standard deviation (SD). A two tailed paired Student’s T-test or two way analysis of variance was performed with Bonferoni correction for multiple comparisons (Graph Pad Prism version 5.04) to compare groups. Correlation was assessed using Pearson coefficient.

## Results

Ten healthy volunteers were recruited. All participants had sputum induction. One subject, however, was unable to tolerate bronchoscopy leaving BALF/TW analysis for the remaining nine participants. The clinical data and summary of the participant characteristics were listed in Table 1.

**Table 1:** Subject characteristics

**Characteristics**	
**Age (Range)**	26 (18–36)
**M:F**	6:4
**FEV**_**1 **_**(L)**	4.22 ± 0.91*
**Weight (kg)**	78 ± 12*
**Choline dose infused (mgs)**	275 ± 54*
**BALF Volume recovery (%)**	42 ±20*
**TW Volume recovery (%)**	31 ± 12*

*M*, Male; *F*, Female; *FEV*_*1*_, Forced expiratory volume in 1 second; *BALF*, Bronchoalveolar lavage fluid; *TW*, Tracheal wash. *Data expressed as mean ± SD.

### Total phospholipid, phosphatidylcholine and SP-D concentrations

The mean total phospholipid concentration in BALF was 64.4 (range 29.1-145.1) nmol/ml, TW 51.8 (range 13.5- 158.7) nmol/ml and IS 8.0 (Range 3.2-14.1) nmol/ml. The total PC concentration in BALF was relatively high (49.9 range 18.6-121.8 nmol/ml) followed by TW (37.2 range 8.5-134.7 nmol/ml) compared to IS (5.8 range 2.4-10.5 nmol/ml). The mean SP-D concentration was 30.3 (range 9.9-67.6) ug/ml for BALF, 24.4 (range 11.8-48.1) ug/ml for TW and 17.5 (range 1.5-85.0) ug/ml for IS. The ratio of total SP-D/PC was much higher for IS, suggesting the possibility of additional secretion of SP-D from airway Clara cells (Table 2).

**Table 2:** Concentrations of total phospholipid, phosphatidylcholine and surfactant protein D

	**BALF**	**TW**	**IS**
**Total PL (nmol/ml)**	64.4 ± 37.1 (29.1–145.1)	51.8 ± 44.6 (13.5–158.7)	†8.0 ± 3.8 (3.2–14.1)
**Total PC (nmol/ml)**	49.9 ± 32.8 (18.6–121.8)	37.2 ± 39.1 (8.5–134.7)	†5.8 ± 2.9 (2.4–10.5)
**SP-D (μg/ml)**	30.3 ± 16.6 (9.9–67.6)	24.4 ± 13.9 (11.8–48.1)	*17.5 ± 25.3 (1.5–85.0)
**SP-D:PC**	0.61	0.66	3.0

*BALF*, Bronchoalveolar lavage fluid; *TW*, Tracheal wash; *IS*, Induced sputum; *PL*, Phospholipid; *PC*, Phosphatidylcholine; *SP-D*, Surfactant protein D. *P < 0.05, ^†^P < 0.01(paired Student’s t-test) when compared with BALF, data expressed as mean ± SD.

### Endogenous lipid compositions

#### ***Phospholipid classes***

**Figure 1:**
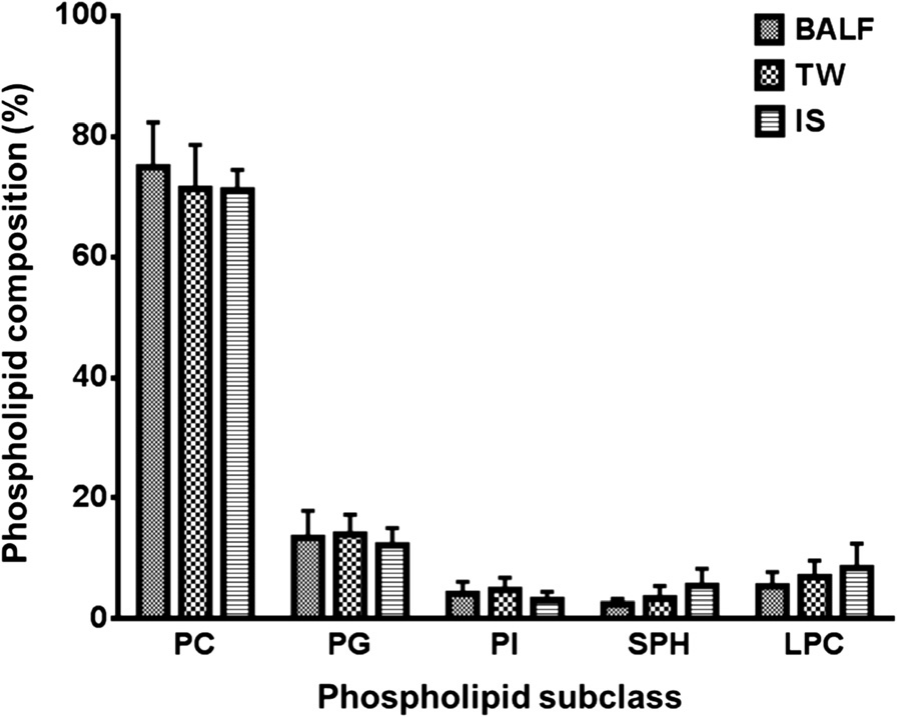
**Fractional composition of phospholipids from bronchoalveolar lavage fluid BALF), tracheal wash (TW) and induced sputum (IS).** PC, phosphatidylcholine; PG, phosphatidylglycerol; PI, phosphatidylinositol; SPH, sphingomyelin; LPC, lyso-phosphatidylcholine. Data expressed as mean ± SD. There was no significant difference in overall phospholipids composition between recovery methods.

The fractional phospholipid composition was investigated by measuring the relative proportions of total PC, PG, PI, SPH and LPC, each determined as the sum of these individual molecular species. Phosphatidylethanolamine (PE) and phosphatidylserine (PS) were present at low concentrations and would have required additional analytical scans to assess molecular composition and consequently, these components are not presented here. PC (75%) followed by PG (13%) were the most abundant phospholipids. Although IS had a fractional increase in LPC and SPH, the phospholipid molecular composition was comparable among all sample types without any statistical difference (Figure 1).

#### ***Molecular composition of phospholipid classes***

ESI/MS enabled a comprehensive analysis of molecular compositions of PC, LPC and SPH using positive and PG and PI with negative conditions. In BALF, di-saturated PC16:0/16:0 was the dominant PC accounting for more than 50% of total PC. This was followed by PC16:0/18:1 (13%), PC16:0/16:1 (9%), PC16:0/14:0 (9%) and PC16:0/18:2 (6%). These findings were consistent with previously published data [9]. When BALF PC composition was compared with other recovery methods, significantly lower proportions of PC16:0/16:0 were noted in both TW and IS. Additionally, IS also had significantly lower proportions of PC16:0/14:0 and PC16:0/16:1 and higher composition of PC16:0/18:1 and PC18:0/18:2 compared to BALF (Table 3). These differences suggest the possibility of dilution of surfactant PC by phospholipids derived from non-alveolar origin in induced sputum.

**Table 3:** Phosphatidylcholine and lysophosphatidylcholine molecular species composition from three isolation methods

**Surfactant PC composition (%)**			
**PC Species**	**BALF**	**TW**	**IS**
PC16:0/14:0	8.8 ±0.9	7.9 ± 1.3	†7.0 ±1.6
PC16:0a/16:0	2.6 ±0.4	2.5 ± 0.4	3.0 ±0.6
PC16:0/16:1	9.0 ±1.5	8.1 ± 1.9	†7.0 ±1.3
PC16:0/16:0	53.3 ± 3.6	^‡^48.7 ± 6.4	^‡^45.8 ±7.6
PC16:0/18:2	5.7 ±1.1	6.9 ±1.8	^†^7.6 ±1.8
PC16:0/18:1	12.6 ±1.6	13.9 ±1.9	^‡^15.3 ±2.4
PC16:0/20:4	1.4 ±0.4	1.8 ±0.8	1.9 ±0.7
PC18:1/18:2	1.6 ±0.5	2.3 ±1.0	2.9 ±0.9
PC18:0/18:2	2.7 ±1.0	4.0 ±2.0	^‡^5.4 ±2.0
PC18:0/18:1	1.3 ±0.6	2.1 ±1.2	2.9 ±1.3
PC18:1/20:4	0.4 ±0.2	0.6 ±0.4	0.5 ±0.2
PC18:0/20:4	0.6 ±0.4	1.2 ±0.9	0.9 ±0.4
**Surfactant LPC composition %**			
**LPC Species**	**BALF**	**TW**	**IS**
LPC16:0	71.7 ±6.0	‡65.7 ±9.2	‡54.4 ±14.8
LPC18:2	6.4 ±1.4	8.0 ±2.3	†12.5 ±5.4
LPC18:1	13.3 ±2.5	15.1 ±2.8	‡21.6 ±6.2
LPC18:0	6.0 ±2.0	8.0 ±3.3	8.3 ±3.7
LPC20:4	2.6 ±1.8	3.2 ±2.1	3.2 ±2.2

*BALF*, Bronchoalveolar lavage fluid; *TW*, Tracheal wash; *IS*, Induced sputum; *PC*, Phosphatidylcholine; *LPC*, Lysophosphatidylcholine. ^†^P < 0.01, ^‡^P < 0.001. Data are expressed as % (mean ± SD) composition of total selected species at time points sampled.

PG molecular species mainly comprised PG16:0/18:1 (35%), PG18:1/18:1 (20%), PG18:0/18:1 (19%) while PI was dominated by PI18:0/18:1 (22%), PI16:0/18:1 (20%) and PI18:1/18:1 (20%). This finding of unsaturated molecular species enrichment among PG and PI species is consistent with previously published data [9,10]. There were no significant differences in PG and PI compositions between BALF and TW. However, small but significant differences were noted in PG16:0/18:1 (3.3% lower, P = 0.01), PG18:1/18:1 (3.1% lower, P = 0.02), and PI18:0/20:4 (4.8% higher, P = 0.02) in IS compared to BALF.

SPH species contain the same phosphocholine head group as PC and consequently can be readily detected by precursor scans of m/z + 184 in ESI/MS. Major SPH species of BALF surfactant composed of SPH16:0 (65%), SPH24:1 (20%) and SPH24:0 (10%). Other minor species (SPH16:1, SPH18:0, SPH18:1, SPH18:2 and SPH20:4) were detected at much lower abundance (<3%). There were no significant differences in SPH molecular composition between BALF and TW. However, significant compositional differences with relative decrease in SPH16:0 (2.9% lower, P = 0.0003) and increase in SPH24:1 (1.8% higher, P = 0.04) was noted in IS compared to BALF.

### Total PC and fractional PC methyl-D_9_ Incorporation

**Figure 2:**
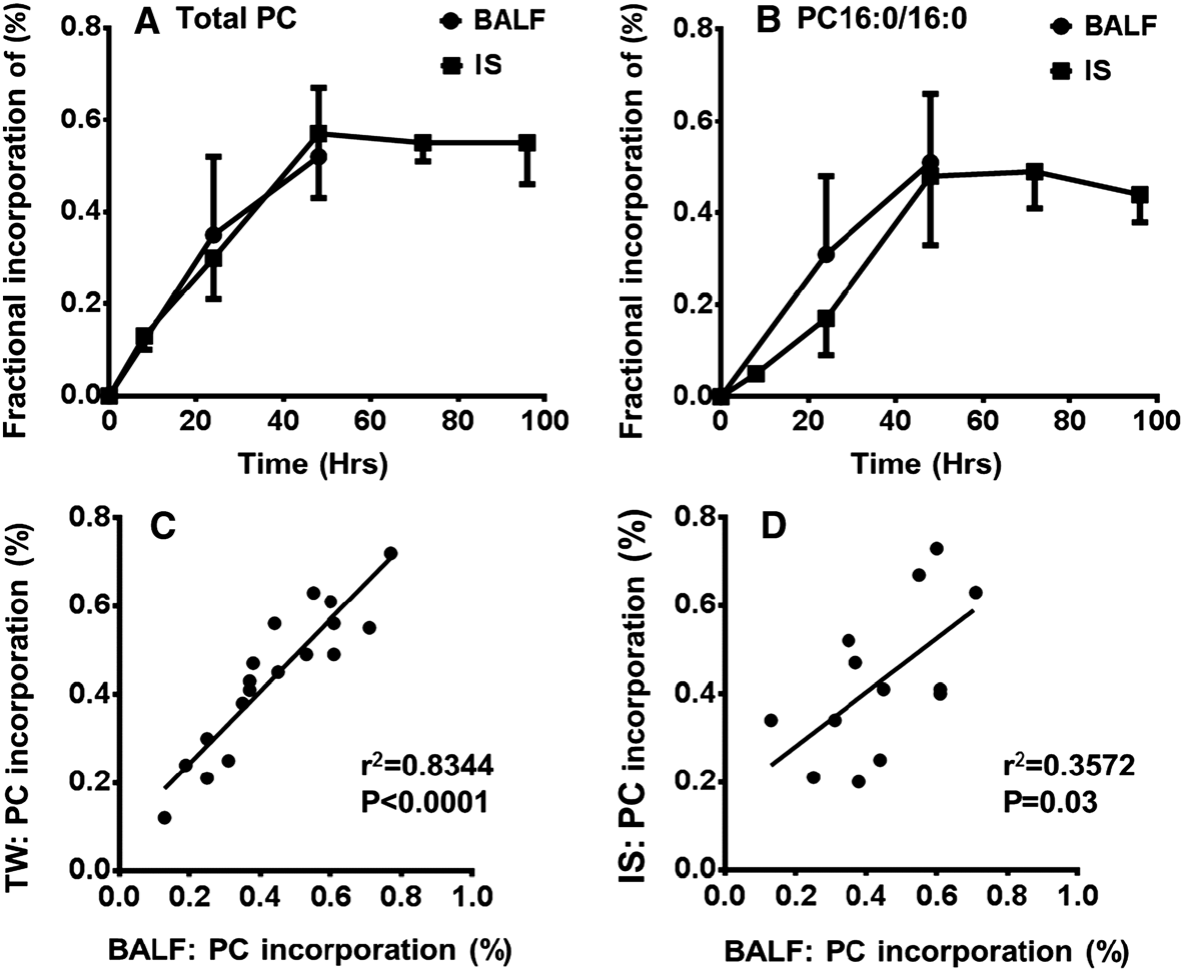
**Fractional incorporation of *methyl-*D**_**9**_**-choline into total phosphatidylcholine (A) and PC16:0/16:0 (B) from bronchoalveolar lavage fluid and induced sputum.** Comparison of *methyl*-D_9_ incorporation into total PC between bronchoalveolar lavage and tracheal wash **(C)** and bronchoalveolar lavage and induced sputum **(D)**. The incorporation data for tracheal wash is not shown here as it followed the exact same pattern as BALF. Results are calculated as from the abundance of *methyl*-D_9_-PC species expressed as a percentage of endogenous PC + *methyl*-D_9_-PC species (mean ± SD. Incorporation into PC16:0/16:0 but not into total PC was significantly lower in IS than BALF at 24 h (*P < 0.05) but not at 48 h. (PC, phosphatidylcholine; BALF, bronchoalveolar lavage fluid; IS, induced sputum, r^2^-Pearson correlation coefficient).

*Methyl*-D_9_-choline incorporation was measured at two time points (24 and 48 hours) for BALF and TW and five time points (8, 24, 48, 72 and 96 hours) for IS. In BALF, the total *methyl*-D_9_-choline incorporation into surfactant PC was 0.35 ± 0.17% at 24 hours and 0.52 ± 0.15% at 48 hours with a rate of incorporation of 0.011 ± 0.002% per hour until 48 hours (Figure 2). There was no difference in total PC *methyl*-D_9_-choline incorporation between BALF and TW. In addition, there was a positive correlation between BALF and TW for both time points (r^2^ = 0.8344, P < 0.0001) for each individuals (Figure 2).

The *methyl*-D_9_-choline incorporation into total PC for IS was 0.13 ± 0.03% at 8 hours, showed a linear incorporation until 48 hours (r^2^ = 0.9984, P = 0.02) at a rate of 0.012 ± 0.0005% per hour and remained relatively stable between 48–96 hours. The fractional incorporation into IS PC16:0/16:0 (0.17 ± 0.08%) was significantly lower (P < 0.05) at 24 h than that into either BALF (0.31 ± 0.17%) or TW (0.29 ± 0.15%) (Figure 2). However, fractional incorporation into IS PC16:0/16:0 reached equilibrium with other sample types at 48 hours. Although there was a positive correlation between BALF and IS for both time points (r^2^ = 0.3572, P = 0.03), this association was much weaker than that for TW and BALF (Figure 2).

### Molecular specificity of D_9_-labelled PC species

**Figure 3:**
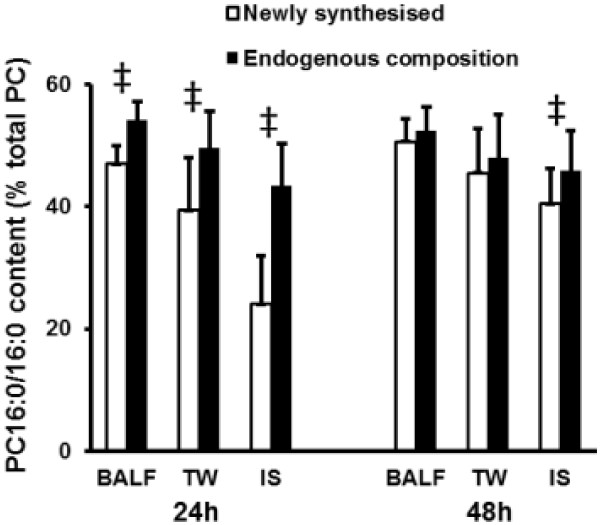
**Molecular specificity of *methyl*-D**_**9**_**-choline incorporation into dipalmitoylphosphatidylcholine.** PC16:0/16:0 synthesis (open bars), expressed as a percentage of newly-synthesized total PC, was compared with that of endogenous PC16:0/16:0 (closed bars) at both 24 and 48 h for broncholaveolar lavage (BALF), tracheal wash (TW) and induced sputum (IS) (mean ± SD; ^‡^P < 0.001 calculated by two way analysis of variance).

The fractional composition of newly synthesized PC varied considerably between sample type and with time. The proportion of newly synthesized PC present as PC16:0/16:0 was consistently lower than that of endogenous PC composition for all sample types at 24 hours (Figure 3). In contrast, at 48 hours only IS still exhibited a lower proportion of newly synthesized compared with endogenous PC16:0/16:0. Inspecting the molecular specificity of PC synthesis in more detail for TW (Table 4), PC16:0/16:0 was the single molecular species significantly different from BALF at both 24 hours and 48 hours. The pattern of newly synthesized PC in IS was very different from BALF, with a total of six significantly different components at 24 hours and still two at 48 hours (Table 4).

**Table 4:** **Fractional composition of methyl-D**_**9**_**-choline incorporation into newly synthesized PC at 24 and 48 hours**

**Newly synthesized D**_**9**_**-choline labelled molecular PC composition at 24 and 48 hours**						
	**24 hours**			**48 hours**		
**PC species (%)**	**BALF**	**TW**	**IS**	**BALF**	**TW**	**IS**
PC16:0/14:0	10.0 ±2.5	7.8 ±2.8	7.9 ±3.2	8.8 ±1.0	7.5 ±1.0	7.3 ±1.9
PC16:0a/16:0	2.6 ±0.6	2.4 ±0.6	4.0 ±2.9	2.3 ±0.5	2.3 ±0.5	3.5 ±0.9
PC16:0/16:1	10.0 ± 2.5	8.9 ±2.4	7.03 ±2.0	9.3 ±1.7	8.1 ±2.1	7.6 ±2.0
PC16:0/16:0	46.9 ±3.0	^‡^39.4 ±8.5	^‡^22.5 ±7.9	50.6 ±3.8	^‡^45.4 ±7.3	^‡^40.4 ±5.8
PC16:0/18:2	6.9 ±1.8	8.32 ±2.7	^†^12.6 ±2.3	6.1 ±1.4	8.0 ±2.4	8.3 ±0.9
PC16:0/18:1	14.5 ±1.7	17.5 ±2.8	*18.2 ±4.5	13.9 ±1.9	14.9 ±1.8	15.9 ±2.2
PC16:0/20:4	1.5 ±0.6	2.2 ±0.9	4.5 ±1.7	1.5 ±0.5	2.0 ±0.7	2.1 ±1.0
PC18:1/18:2	1.9 ±0.7	3.1 ±1.5	*5.8 ±1.9	2.0 ±0.6	2.7 ±0.9	3.5 ±1.4
PC18:0/18:2	3.2 ±0.9	5.4 ±2.8	^‡^9.1 ±3.0	2.8 ±1.1	4.6 ±2.3	*6.0 ±1.9
PC18:0/18:1	1.3 ±0.6	2.5 ±1.2	*4.7 ±2.3	1.5 ±0.8	2.3 ±1.8	3.3 ±1.1
PC18:1/20:4	0.5 ±0.2	0.9 ±0.5	1.5 ±1.1	0.4 ±0.2	0.7 ±0.4	0.9 ±0.4
PC18:0/20:4	0.7 ±0.4	1.7 ±0.9	2.1 ±0.9	0.7 ±0.5	1.3 ±1.1	1.1 ±0.4

*BALF*, Bronchoalveolar lavage fluid; *TW*, Tracheal wash; *IS*, Induced sputum; *PC*, Phosphatidylcholine. *P < 0.05, ^†^P < 0.01, ^‡^P < 0.001 for TW and IS compared with BALF calculated by two way analysis of variance, data presented as mean ± SD.

### LPC – Composition and methyl-D_9_-choline incorporation

**Figure 4:**
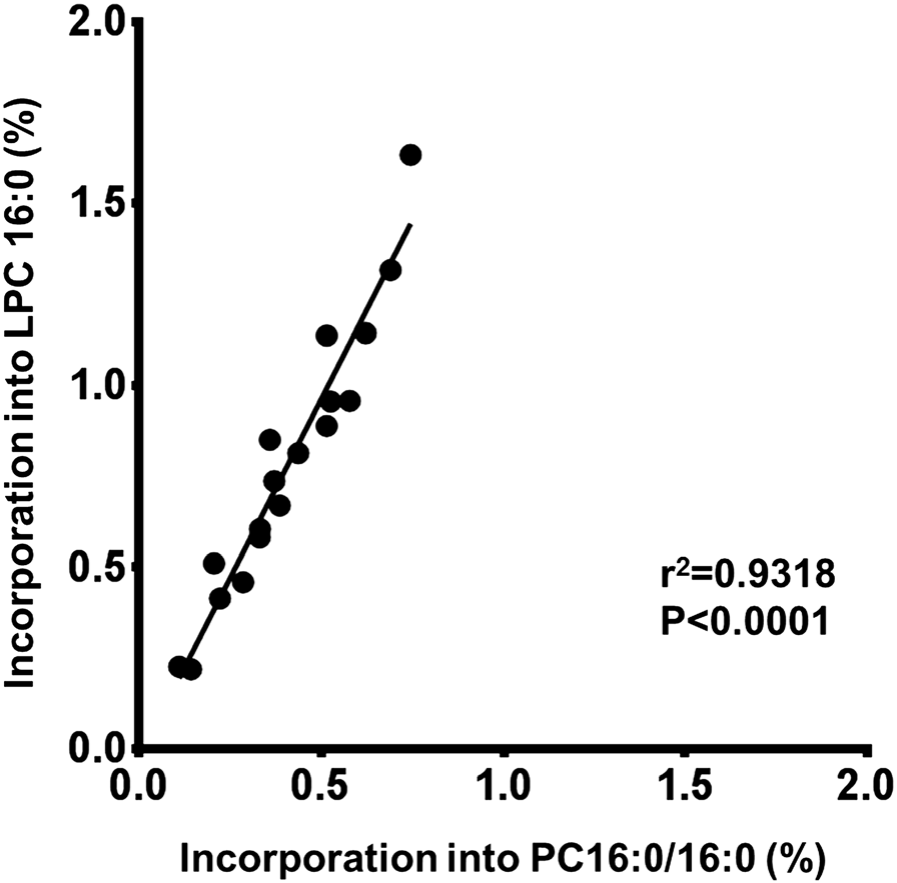
**Comparison between *methyl*-D**_**9 **_**incorporation into PC16:0/16:0 and LPC16:0 isolated by bronchoalveolar lavage.** (r^2^- Pearson correlation coefficient).

The LPC composition mainly consisted of LPC16:0 (72%), LPC 18:1 (13%) and LPC18:2 (6%) (Table 3). Overall total LPC *methyl*-D_9_-choline incorporation was nearly twice than total PC incorporation for both time points (24 and 48 hours). Furthermore, LPC16:0 *methyl*-D_9_-choline incorporation was twice as that of *methyl*-D_9_-labelled PC16:0/16:0 and correlated positively (r^2^ = 0.9318, P < 0.0001) at both time points (Figure 4). However, the LPC 16:0 *methyl*-D_9_-labelling was much lower (~50%) compared to other LPC species.

## Discussion

This study demonstrates for the first time the molecular compositions of various bronchoalveolar compartments in human model and the surfactant molecular PC kinetics from all these compartments. The results showed that surfactant extracted by TW closely resembled that of BALF. As ATII cells are the only source of pulmonary surfactant phospholipids, the significant positive correlation of *methyl*-D_9_-choline incorporation between BALF and TW suggests the mucocillary transit time of surfactant up along the airways had little significant impact on surfactant PC kinetics. Our analysis suggests that, despite the presence of already-secreted surfactant lining the small and large airway epithelium, a surfactant pool accessible from the upper airways by tracheal wash is in isotopic equilibrium with the alveolar surfactant pool.

Induced sputum had variable phospholipid composition and kinetics compared to BALF, possibly due to several reasons. Firstly, IS phospholipids may represent pools of surfactant with different temporal origins, with some material newly secreted from the alveolus combined with previously secreted surfactant embedded within the mucus layer. Secondly, sputum induction is a complex process and the exact origin of the induced sputum is not always known. Furthermore, the contamination from saliva and other non- surfactant phospholipids may have interfered with surfactant assessment. Although IS had qualitatively similar composition to that of alveolar surfactant [10], it may not be an ideal model to study alveolar surfactant metabolism. Thirdly, the relatively high content of sphingomyelin in induced sputum suggests proportion of IS phospholipid may be of cellular not surfactant origin. Such a cellular origin of IS phospholipid may become more significant in disease states characterized by increased airway inflammatory cell infiltration.

Surfactant PC is synthesized *de-novo* by the CDP-choline pathway. However, about 50% of PC16:0/16:0 is produced by acyl-remodelling mechanisms catalysed by the sequential actions of phospholipase-A_2_ and lysophosphatidylcholine acyltransferase activities [11]. It is widely assumed that surfactant PC synthesis and acyl-remodelling processes precede subsequent secretion [11]. However, our finding challenges this established concept. If the surfactant PC is secreted after acyl-remodelling and maturation process, the secreted surfactant PC16:0/16:0 compositions should reflect the endogenous composition at all time points. Our study shows that the proportion of *methyl*-D_9_-labelled PC16:0/16:0 is equilibrated with endogenous composition only at 48 hours. This implies that a certain proportion of PC is secreted even before the acyl-remodelling mechanisms are complete.

This is the first to study to demonstrate the feasibility of assessing surfactant lyso-PC metabolism *in-vivo* in human subjects. This study shows that LPC16:0 is the principle lyso-PC in pulmonary surfactant, but that total LPC *methyl*-D_9_ incorporation was twice as that of total PC. The *methyl*-D_9_-incorporation of LPC16:0 is much higher than that of PC16:0/16:0 at both 24 and 48 hours. This finding challenges the paradigm of that the LPC is a formed by hydrolysis of secreted surfactant PC. The much higher fractional incorporation of LPC compared to PC precludes LPC coming from hydrolysis of newly synthesized PC and emphasizes the complexity of the underlying mechanisms of surfactant synthesis and secretion. These observations strongly support the possibility, at least in healthy individuals, that LPC is secreted together with the other surfactant phospholipid rather than simply being a consequence of hydrolysed PC. While phospholipase-mediated hydrolysis of secreted surfactant phospholipid remains a possibility in inflammatory lung disease, a further implication of our results, supported by the strong correlation of stable isotope label incorporations into LPC16:0 and PC16:0/16:0 (Figure 4) is that secreted LPC may be ultimately a consequence of acyl remodelling mechanisms within ATII cells.

Investigators have also used other stable isotopes such as ^13^C-glucose, ^13^C-fatty acids or deuteriated water to assess fractional synthetic rates (FSR) of saturated PC (SatPC) to characterise surfactant kinetics in neonates [12-14] and adults [15]. SatPC is the major PC surfactant component and a clinical marker of lung injury and surfactant derangement [16]. Measuring SatPC from deuteriated water enables maintenance of a steady state condition more easily, and therefore results can be compared between groups of patients with different clinical conditions. These novel studies provided significant insights into surfactant metabolism in humans but have some limitations. First, these methodologies initially used osmium tetroxide oxidation of unsaturated phospholipid to generate a SatPC fraction. Consequently, minimal information can be obtained about the molecular specificity of surfactant PC metabolism [17]. Second, the laborious sample preparation required for these studies has precluded the early diagnostic application of these methodologies to individuals with lung diseases. In contrast, the use of ESI-MS to monitor stable isotope incorporations requires minimal sample preparation and has the potential to generate diagnostic surfactant kinetic results in a time scale consistent with making clinical treatment decisions for individual patients with acute respiratory compromise.

In our study, tracheal secretions were accessed by the use of a bronchoscope. It is entirely plausible that a small amount of the BALF from distal alveolus may have contaminated the tracheal washings as both of these procedures were conducted in a single setting. If the compositional similarities were due to this contamination, one might have seen significant dilution effect in the magnitude of 1:10 reduction in the fractional phospholipid concentrations from tracheal wash. However, this was not the case and this absence of dilution effect indicate any contamination of tracheal wash from alveolar material is minimal.

Another limitation of this study was the measurements of absolute amounts of phospholipid subclasses and PC species may have been influenced by the variability in the sample recovery. Nevertheless it was reassuring to see overall compositional similarities from all endobronchial sampling. Furthermore, kinetic data regarding *methyl*-D_9_ incorporation patterns are unlikely to be influenced by this variability in recovery as the incorporation data was corrected for the endogenous material.

Acute lung injury and ARDS are characterized by significant quantitative and qualitative alterations in surfactant phospholipids composition [5]. Despite these findings, therapeutic attempts with exogenous surfactant remain unhelpful in this population [18]. The complexity of ARDS pathogenesis suggests several possible mechanisms for surfactant dysfunction, such as impaired synthesis and secretion, increased hydrolysis, proteolysis and oxidation or functional inhibition by infiltrating plasma proteins. However, in-vivo human models investigating such complex underlying mechanisms are lacking. Deficient understanding of the potential multiple mechanisms of aberrant surfactant metabolism in this disease cohort may in part explain the lack of anticipated clinical benefits from exogenous surfactant replacement strategies. Application of stable isotope studies in this population may possibly identify underlying phenotypes to characterise patients according to pathological mechanisms of surfactant dysfunction. Tracheal washings substituted for quantitative bronchoalveolar lavage may be an alterative for surfactant isolation in patients otherwise unable to tolerate invasive procedures without clinical compromise.

## Conclusions

This study comprehensibly demonstrates the feasibility of performing stable isotope labelling to study surfactant phospholipid kinetics from bronchoalveolar compartments in healthy adults. Tracheal secretions were more closely resembled alveolar surfactant composition compared to induced sputum. Although several differences were noted in induced sputum phospholipid composition and turnover compared to BALF, the overall relative changes were small. This study illustrates the utility of various recovery methods to study *in-vivo* surfactant metabolism in humans which can be applied in disease states such as ARDS to possibly identify variation in surfactant metabolism among patients.

## Abbreviations

ALI: Acute lung injury; ARDS: Acute respiratory distress syndrome; ATII cells: Alveolar type II cells; BALF: Bronchoalveolar lavage fluid; ESI-MS: Electrospray ionisation mass spectrometry; IS: Induced sputum; LPC: Lysophosphatidylcholine; PC: Phosphatidylcholine; PG: Phosphatidylglycerol; PE: Phosphatidylethanolamine; PI: Phosphatidylinositol; PS: Phosphatidylserine; SatPC: Saturated phosphatidylcholine; SPH: Sphingomyelin; TW: Tracheal wash.

## Competing interests

ADP has no direct financial interest in the work presented in this study; his surfactant research programme is supported by kind donation of a therapeutic surfactant from Chiesi for a clinical trial. The remaining authors declare that they have no competing interests.

## Authors’ contributions

AD co-ordinated the study, contributed to the study design and the clinical and laboratory procedures, calculated the results and drafted the manuscript; VG co-ordinated the laboratory procedures and contributed to data interpretation and manuscript revision; RC contributed to the study design, ethics application, clinical procedures and manuscript revision; MPWG contributed to the study design, clinical procedures and manuscript revision; ADP contributed to the study design, data interpretation and manuscript revision. All authors read and approved the final manuscript.

## Pre-publication history

The pre-publication history for this paper can be accessed here:

http://www.biomedcentral.com/1471-2466/14/10/prepub
